# Role of c-Kit in Myocardial Regeneration and Aging

**DOI:** 10.3389/fendo.2019.00371

**Published:** 2019-06-19

**Authors:** Fabiola Marino, Mariangela Scalise, Eleonora Cianflone, Teresa Mancuso, Iolanda Aquila, Valter Agosti, Michele Torella, Donatella Paolino, Vincenzo Mollace, Bernardo Nadal-Ginard, Daniele Torella

**Affiliations:** ^1^Molecular and Cellular Cardiology, Department of Experimental and Clinical Medicine, University Magna Graecia, Catanzaro, Italy; ^2^Department of Health Sciences, Interregional Research Center on Food Safety and Health (IRC-FSH), University Magna Graecia of Catanzaro, Catanzaro, Italy; ^3^Interdepartmental Center of Services (CIS) of Genomics, Department of Experimental and Clinical Medicine, University Magna Graecia, Catanzaro, Italy; ^4^Department of Cardiothoracic Sciences, University of Campania L. Vanvitelli, Naples, Italy; ^5^Department of Experimental and Clinical Medicine, University Magna Graecia, Catanzaro, Italy; ^6^StemCell OpCo, Madrid, Spain

**Keywords:** c-kit, cardiac stem cells, cardiac aging, cardiac regeneration, cardiac remodeling

## Abstract

c-Kit, a type III receptor tyrosine kinase (RTK), is involved in multiple intracellular signaling whereby it is mainly considered a stem cell factor receptor, which participates in vital functions of the mammalian body, including the human. Furthermore, c-kit is a necessary yet not sufficient marker to detect and isolate several types of tissue-specific adult stem cells. Accordingly, c-kit was initially used as a marker to identify and enrich for adult cardiac stem/progenitor cells (CSCs) that were proven to be clonogenic, self-renewing and multipotent, being able to differentiate into cardiomyocytes, endothelial cells and smooth muscle cells *in vitro* as well as *in vivo* after myocardial injury. Afterwards it was demonstrated that c-kit expression labels a heterogenous cardiac cell population, which is mainly composed by endothelial cells while only a very small fraction represents CSCs. Furthermore, c-kit as a signaling molecule is expressed at different levels in this heterogenous c-kit labeled cardiac cell pool, whereby c-kit low expressers are enriched for CSCs while c-kit high expressers are endothelial and mast cells. This heterogeneity in cell composition and expression levels has been neglected in recent genetic fate map studies focusing on c-kit, which have claimed that c-kit identifies cells with robust endothelial differentiation potential but with minimal if not negligible myogenic commitment potential. However, modification of c-kit gene for Cre Recombinase expression in these Cre/Lox genetic fate map mouse models produced a detrimental c-kit haploinsufficiency that prevents efficient labeling of true CSCs on one hand while affecting the regenerative potential of these cells on the other. Interestingly, c-kit haploinsufficiency in c-kit-deficient mice causes a worsening myocardial repair after injury and accelerates cardiac aging. Therefore, these studies have further demonstrated that adult c-kit-labeled CSCs are robustly myogenic and that the adult myocardium relies on c-kit expression to regenerate after injury and to counteract aging effects on cardiac structure and function.

## Introduction

In developed countries, modern and up-to-date guidelines-recommended treatments based on solid clinical and basic cardiovascular research have significantly reduced the mortality for acute cardiovascular (CV) syndromes (1, 2). However, the improvement in primary treatment of cardiovascular syndromes bargained a steep surge of patients with chronic heart failure (CHF), a syndrome that nowadays has numbers similar to an epidemy and takes the highest toll on human lives among CV diseases (3).

Indeed, during acute life-saving interventions, most patients irreversibly develop myocardial injury, from which CHF develops. CHF has no available curative therapies and the prognosis for patients is poorer than that for most cancers, having an average survival of only 3–5 years after its onset (1–3). There are almost 40 million patients worldwide with HF that account for a significant part of the annual hospital admissions and that absorb several billions of dollars to the USA healthcare. Similar number of patients and annual costs are emerging to be found in the EU healthcare systems after several statistical analysis (1, 2). It follows that HF treatments currently in use are only symptomatic if not just palliative when considering mortality as main endpoint—with heart transplant as only valid yet practically un-available solution to overcome it. It is imperative indeed to develop technologies to better understand and to monitor CV diseases, their symptoms and complications, with the aim to preserve/enhance the function of the surviving cardiomyocytes, while also to replace the lost cardiomyocytes, primary causes of CHF (1, 2).

Myocardial infarction, and ischemic heart disease in general, is the primary etiology of CHF (1, 3). Also in the cases of the structural cardiomyopathies, where the CHF is of non-ischemic origin, the primary issue is the lack of the myocardium to undergo a robust cardiomyocyte replacement (2). On surprisingly, therefore, regenerative biology/medicine has raised with the goal to find an effective and broadly available therapy to refresh the contractile muscle cells lost and/or permanently dysfunctional in consequence of the primary injury (2, 4). Unfortunately, the predominant skepticism about the intrinsic endogenous regenerative capacity of the adult mammalian heart, including the human have produced often contradictory approaches to perform myocardial repair/regeneration (2).

Until sufficient scientific data are obtained to eventually overcome this widespread skepticism, whereby hard clean and clear data remove the need for interpretations and opinions, no clinical repair or regeneration protocol will be ever able to answer the question of whether it is feasible to functionally regenerate the failing human heart (2).

## Biology of the Adult Heart: the Old Paradigm

The adult cardiomyocytes (CMs), terminally differentiated cardiac parenchymal cells, permanently withdrawn from the cell cycle with no capacity to replicate, have been classically defined as *elementi perenni*, similarly to neurons, and thus believed to last a lifetime (2, 5, 6). The main underlying and ensuing biologic dogma was and still practically remains that, when the heart is subjected to a prolonged work overload or to a diffuse and/or segmental injury, the CMs respond increasing their size, becoming hypertrophic to accommodate a larger number of its sarcomeres to sustain the increased work or just die (2).

This static view of the biology of the adult hearts postulates that from cradle to grave no new CMs are therefore added and it turns that to maintain an equilibrium for the heart to properly function and sustain the systemic circulation throughout life, CM death is a rare, if not negligible, event (2). Thus, under this dogmatic view, post-natal life of the heart is not ruled by a cell homeostasis process where cardiac muscle cells die and are consequently replaced in response to wear and tear and/or injury (2).

On this basis, one of the first attempt, still ongoing, to obtain cardiac muscle regeneration has been and continues to be the re-activation of mitotic division of mature terminally differentiated CMs (7). However, genetic modification of the myogenic differentiation network and muscle cell identity of adult CMs to force their division to produce a robust number of new CMs has mainly resulted in increased polyploidy and/or death, both *in vitro* and *in vivo* (2, 7–9). On the other hand, experimental approaches conducted in order to increase CM division, which have been proven to foster beneficial functional *in vivo* effects (9, 10), are not necessary to clearly rule out whether the detected new cardiomyocyte formation is the product of the division of pre-existing terminally differentiated CM or of myocyte progenitors before their terminal differentiation (2). Moreover, the heart is the organ of the adult human body less affected by neoplastic transformation (11), which has been classically referred to the “stubborn” terminally differentiated state of the adult CMs. It logically turns that the inhibition and/or removal of the CM inhibitory cell cycle checkpoints maintaining their differentiated state in the adult heart in the myocardium will run the high risk of breaking the intrinsic protection of the adult heart from neoplastic development (2).

Overall, the classic dogma of the biology of the adult heart considered nil the regenerative potential of the adult myocardium and its response to increased workload limited to CM hypertrophy. Under these biologic tenants, no effective protocol for myocardial regeneration could be developed unless exogenous effective regenerative agents were discovered and applied. Cardiovascular therapeutic research has been developed under this biologic umbrella up to today (2).

## Biology of the Adult Heart: The New Paradigm

The historic paradigm of mammalian CM terminal differentiation and permanent withdraw from the cell cycle (2, 5–7, 12) started to be challenged by the evidence arising from few reports of sporadic new CM formation in the normal and pathological adult heart (2, 13, 14). As the number of this new CM formation was very small, and it had no biological basis to be mechanistically interpreted, they were disregarded as a curiosity or just an experimental artifact with no physiological significance (2).

The initial yet largely ignored detection of new CM formation in the adult mammalian heart has been recently confirmed and undoubtedly proven by cutting-edge molecular and genetic tracking techniques that have nowadays established that new CMs are continuously born in the post-neonatal mammalian heart, including the human (2, 15–20). However, despite this evidence, the quantification of this CM renewal in the adult heart remains highly debated and it is still widely regarded as a neglegible and therefore physiological useless phenomenon (2, 20). In adult healthy humans, using radioactive isotope decay, an annual CM turnover rate of ~0,5% has been reported through mathematical extrapolation (16, 21). In small mammals, the estimated range of CM annual turnover spans from 0.001 to 4%. Nevertheless, the reliability of all these estimates remain questionable simply because they are extrapolations and not diresct experimental measurements (2).

Nevertheless, while there is a lack of agreement about CM turnover rates, and myocardial regenerative response in general, there is a consensus that the heart response to damage is not sufficient to counteract the CM loss and dysfunction after myocardial infarction (MI) and in CHF (2). Because replacement of lost and injured CMs will continue to call for effective regenerative protocols, it is mandatory for the cardiovascular research community to define an experimental protocol that can directly and accurately quantify CM turnover in health and disease. Nonetheless, the undisputed existence of an intrinsic regenerative response with new CM formation in the adult myocardium is a solid basis to continue the search for its precise nature with the logical expectation that mastering its underlying mechanisms will provide new solutions to develop clinically meaningful protocols of myocardial protection, repair and/or regeneration (1, 2).

## Adult c-kit^pos^ Cardiac Stem Cells: Retracing the Stages of their Discovery

A main obstacle hampering progress toward the development of effective cardiac regeneration protocols remains the lack of consensus about the origin and number of CMs which are born after the early post-natal period [(5, 6) days in the mouse, ~1 year in the human], when heart growth by CM replication stops and all CMs become terminally differentiated.

Since at least 2003, we have known that the mammalian heart, including the human, contains a pool of resident tissue-specific cardiac stem/progenitor cells, the endogenous CSCs (hereafter eCSCs when in the myocardium and CSCs when isolated and studied *in vitro*) (20, 22). Originally, the eCSCs have been identified as a small cardiac cell population through the expression of specific membrane markers, in particular the stem cell factor (SCF) receptor kinase c-kit (23), Sca-1 (24), and MDR-1 (25). *In vitro* and *in vivo* experimental tests have clearly shown that CSCs have all the characteristics expected from a tissue-specific stem cell: they are clonogenic, self-renewing and multipotent. They are indeed able to differentiate *in vivo* and *in vitro* into the main myocardial cell types –cardiomyocytes, endothelial and vascular smooth muscle cells and connective tissue cells (20).

Nevertheless, since the first report and despite a burgeoning and reproducible evidence characterizing tissue specific CSCs, several reports have questioned their existence (26–28). Unfortunately, oversimplification of the available data created the confusion whereby a single marker, i.e., c-kit and then Sca-1 in mice, became more important than the complex and exhaustive experimental approach used in the first place to prove that the heart harbors *bona fide* adult cardiac progenitor cells. Indeed, the experimental evidence that the adult heart contains a pool of cells that are clonogneic, self-renewing and multipotent was swiftly reduced, without an inch of supporting data, to a common notion that cardiac c-kit (or Sca-1) cells are the CSCs. On this basis, it was reported that c-kit^pos^ cells are robustly cardiogenic in the neonatal period while adult c-kit^pos^ cardiac cells are marginally, if at all, myogenic (29). It was also correctly shown that c-kit^pos^ cardiac cells are endothelial cells or mast cells, but this data, unsurprising because known since decades, was used to claim that c-kit^pos^ CSCs were just mast cells in the adult human heart (26–28). Despite the latter negative reports, after the first identification of eCSCs in the adult rodent heart (22), different groups have independently proven the existence of cells with similar characteristics and regenerative potential in practically all the mammalian species, including the human (30–37). Interestingly, the first report of the existence of cardiac tissue specific progenitors from human tissue was obtained by Messina et al. (32). These authors reported the isolation of undifferentiated cells that grow as self-adherent clusters (termed “cardiospheres”) from subcultures of post-natal cardiac human biopsy specimens and also from murine hearts. These cells are clonogenic, having the properties of adult cardiac stem cells. Indeed, they are capable of long-term self-renewal and can differentiate *in vitro* and after transplantation in SCID beige mouse in cardiomyocytes and vascular cells (32). Cardiospheres appear to have a bone marrow origin (38). c-kit^pos^ human CSCs (hCSCs) have been then isolated by explant culture technique and enzymatic digestion from myocardial samples of the four cardiac chambers of patients with ischemic and non-ischemic cardiomyopathy (39). These c-kit^pos^ hCSCs are self-renewing and clonogenic, and their capacity to generate clones from a single cell appears to be similar to their rodent counterparts. All the hCSCs clones tested express sizable levels of c-kit and they are negative for both hematopoietic and endothelial markers. When grown in suspension, these cells are able to form cardiospheres and, under adequate stimuli, they differentiate *in vitro* into cardiomyocytes, vascular smooth muscle and endothelial cells. Also hCSCs support myocardial regeneration when injected in immunodeficient rats with myocardial infarction. Further data on c-kit^pos^ cardiac cells have been obtained through c-kit^BAC^-EGFP transgenic mice, in which EGFP expression is placed under control of the c-kit locus (40, 41). These reports showed that the myocardial c-kit-EGFP^pos^ cells increases in early post-natal growth, but declines in the first weeks after birth (40, 41).

c-kit-EGFP^pos^ cells isolated from neonatal hearts commit to all three cardiac lineages and, after plating in appropriate cardiac differentiation media, many c-kit-EGFP^pos^ cells differentiate into spontaneously contracting cells (40). When adult c-kit^BAC^-EGFP^pos^ mice underwent coronary ligation to produce myocardial infarction, it was shown that c-kit expression increased significantly at 7 days after injury and declined by 4 weeks to baseline levels. Modest c-kit-EGFP^pos^ expression was observed in striated mature cardiomyocytes in the border zone (40). On the contrary, using a different approach, Fransioli et al. show elevated c-kit expression in the infarcted and border regions throughout 10 days after injury (41). Remarkably, they found c-kit-EGFP^pos^ cell recruitment to the area of injury, with their differentiation into cardiomyocytes, smooth muscle and endothelium (41).

In 2011 we showed that the adult pig myocardium, a frequently used and widely accepted pre-clinical large animal model for cardiac disease, harbors among the c-kit-labeled cells a significant fraction of blood-committed CD45^pos^ cells. On the contrary, c-kit^pos^/CD45^neg^ pig cardiac cells behave as cardiac tissue specific stem/progenitor cells. These c-kit^pos^/CD45^neg^ CSCs can be activated to proliferate when exposed to *in vitro* treatment with insulin-like growth factor (IGF)-1 and hepatocyte growth factor (HGF), while in differentiation conditions, they commit to cardiomyogenic lineage when treated with a combination of IGF-1 and HGF (42). These *in vitro* data was the basis to pre-clinical test of the intracoronary injection of small amounts of IGF-1 and HGF (a single dose ranging from 0.5 to 2 μg HGF and 2 to 8 μg IGF-1) to pigs subjected to acute myocardial infarction (AMI) by transient coronary occlusion. This experimental approach produces a robust activation of the eCSCs pool with ensuing robust cardiac muscle regeneration and cardiac function improvement (42).

The intracoronary IGF-1+HGF cocktail, in a dose-dependent manner, boosted myocardial regeneration but also it improved cardiomyocyte survival, and reduced both fibrosis and cardiomyocyte reactive hypertrophy. A single administration of IGF-1/HGF was sufficient to have a pronounced and durable beneficial effect, because through a paracrine effect on the endogenous myocardium activated a feedback loop on the targeted cells for the their production of cardiopoietic growth and survival factors. The histological changes correlated with a reduced infarct size and a better ventricular segmental contractility and ejection fraction when compared to control animals as assessed by cMRI (42). Similar positive effects were obtained when the IGF-1/HGF combination was administered trans-endocardially in pigs with a chronic MI using the NOGA system (43). Despite its effectiveness, the administration of IGF-1/HGF is insufficient for complete cellular maturation of the newly-formed CMs. Despite the beneficial effect of the therapy in reducing the scar area, pathological remodeling, and partial recovery of ventricular function, the growth factor combination must be still refined to include an improved cocktail that can generate a more rapid recovery of the ventricular mass capable to sustain a proper adult myocardium force.

Subsequently, to directly assess the endogenous regenerative potential of eCSCs we made use of a severe diffuse myocardial damage in the presence of a patent coronary circulation produced by high doses of Isoproterenol (ISO) that, unlike the segmental myocardial loss produced by permanent coronary ligation, spares the eCSCs (44). CSCs in culture are resistant even to the highest doses of ISO, which at the contrary kills the majority of primary cardiomyocytes at significantly lower doses (44). This resistance of CSCs to ISO is equally evident in animals *in vivo* so that eCSCs are available to respond to CM loss by the ISO-induced myocardial injury showing their regenerative endogenous potential. Using this experimental setting we have provided for the first time the evidence that eCSCs spontaneously and completely replace all the CMs lost following diffuse extensive myocardial damage which has killed ~10% of the ventricular myocytes. If the eCSCs are ablated, through the administration of the antimitotic agent 5-flurouracil (5-FU), there is absence of CM replacement and the lost contractile mass triggers terminal HF. If failing hearts devoid of their eCSC pool are treated with adoptive transfer of exogenous CSCs, progeny of a single syngeneic CSC, the endogenous CSC deficiency is corrected, and CM deficit is filled up with new CMs derived by the differentiation of the transplanted CSCs. This CSC-dependent regenerative process returns the myocardium to the *status quo ante*, the tissue damage is repaired reverting HF and normal cardiac function is restored. Subsequent selective ablation of these transplanted and engrafted CSCs and their differentiated progeny obtained by genetic activated suicide, rapidly sets the heart back in overt HF followed by death unless a new batch of CSCs is transplanted (45). Thus, using a variety of well-accepted genetic, cellular and molecular approaches we have provided the first evidence that the c-kit^pos^ eCSCs are necessary and sufficient for myocardial cell regeneration (45). Clearly this evidence is proof of concept because it does not question that this regenerative potential of the heart is inadequate to counteract the segmental loss by myocardial infarction.

Of note, to track c-kit^pos^ CSC fate *in vivo*, we generated a c-kit/Cre construct containing a short 5′ flanking region (~0.6 kb) of the c-kit promoter including the transcription initiation site (TIS) and the HS1 and HS2 sequences, important for cell specific expression (46) and Cre recombinase cassette inserted in frame with the ATG site within the exon 1 of c-kit. We demonstrated that this transgenic construct when released intra-myocardially through a lentiviral vector is not re-expressed either prior or after ISO injury in adult CMs (45). Moreover, when a similar c-kit/EGFP construct was injected into wild type mice, it correctly labeled c-kit interstitial cardiac cells including c-kit^pos^ CSCs but no mature CMs either in normal hearts or after ISO injury (45). Thus, this set of experiments were able to faithfully track the c-kit^pos^CSC fate *in vivo* after ISO establishing their robust cardiomyogenic potential.

## Phenotypic Identity of True Endogenous Adult Cardiac Stem Cells: c-kit Expression Is Necessary but not Sufficient for their Identification

The wrong notion that a single marker on its own, as c-kit, can identify a population of CSCs (47–51) has been the basis for apparently negative data about the nature and regenerative capacity of the “c-kit^pos^ cardiac cells” (47–51), which has created a significant and widespread skepticism on the validity of genuine data. It is worth noting here that the identification of a cell population expressing c-kit or whatever other single marker is clearly insufficient to score such cell pool as an homogenous c-kit positive stem cell population (52, 53). The adult heart contains a heterogeneous mixture of c-kit^pos^ cardiac cells. These c-kit-expressing cardiac cells are mainly mast and endothelial cells (52). Specifically, a myocyte-depleted preparation of pure c-kit^pos^ cardiac cells contains more than 90% of cells expressing blood and endothelial lineage markers, such as CD45 and CD31 (Lin^pos^). These lineage-committed c-kit-labeled cells are not myogenic progenitors and do not possess stem cell properties *in vitro* and *in vivo* (52, 54). On the contrary, 10% of the total c-kit-expressing cardiac cells are negative for all the lineage-committed markers, including CD45 and CD31 among others. This c-kit^pos^CD45/CD31^neg^ (also referred as lineage negative) cardiac cell population is enriched with a yet incompletely phenotypic-defined cell population that shows the prototypical stem cell properties *in vitro*: i.e., multipotent, self-renewal and clonogenesis (52, 54). Thus, to identify and isolate true multipotent CSCs is essential to eliminate, from the total c-kit-expressing cardiac cells, the most abundant lineage-committed (Lin^pos^) cells. A negative sorting with CD45/CD31 antibodies followed by a c-kit antibody positive selection allows to obtain a cardiac cell population that is negative for CD45, CD31, and CD34 (Lin^neg^) (52–54) and positive yet in different percentages for Sca-1, Abcg2, PDGFR-α, Flk-1, MDR-1, and CD166, all markers previously used by different groups to isolate endogenous resident cardiac cells with progenitor potential. Ten percent of the CD45^neg^CD31^neg^c-kit^pos^ cardiac cells population are clonogenic and are able to differentiate, *in vitro* and *in vivo*, into mature and functional CMs as well as vascular cells (52–57). A small fraction of freshly isolated CD45^neg^CD31^neg^c-kit^pos^ cardiac cells express pluripotency genes (Oct-4, Nanog, Klf-4, and Sox-2), but also stemness regulatory genes and typical transcription factors of the early stages of cardiac myogenic differentiation (Tert, Bmi-1, Gata-4, Mef2c, and Nkx2.5) (58–62). On the other hand, the lineage positive (CD45^pos^ and CD31^pos^), Lin^pos^c-kit^pos^ cardiac cells are negative for all these mutipotency genes and myogenic transcription factors and are solely able to differentiate into endothelial cells (54).

To narrow down the phenotype of true multipotent CSCs we obtained several clones from deposition of single CD45^neg^CD31^neg^c-kit^pos^ cardiac cells. These cloned and sub-cloned Lin^neg^c-kit^pos^ CSCs homogenously maintain a stable phenotype without signs of growth arrest, senescence or down regulation of stemness and cardiac gene expression. When grow in suspension, cloned Lin^neg^c-kit^pos^ CSCs generate cardiospheres, and when were placed in established differentiation media for cardiomyocyte, smooth muscle and endothelial cell lineages, they differentiate into CMs, smooth muscle and endothelial cell lineages, respectively, at a significantly higher rate compared with the freshly isolated CD45^neg^CD31^neg^c-kit^pos^ cardiac cells (52, 54). The Lin^neg^c-kit^pos^ cloned CSCs uniformly express c-kit, PDGF-Rα, CD166, SSEA-1, Nestin, Bmi-1, Tert, Gata-4, and Nkx2.5 and are negative for CD34, CD45, and CD31. All cloned CSCs also uniformly express the pluripotency genes Oct3/4, Nanog, Klf-4, and Sox-2 (22, 52–54, 63, 64). Thus, these experiments on single CD45^neg^CD31^neg^c-kit^pos^ cardiac cell-derived CSC clones prospect the phenotypic identity of the true endogenous CSC.

Cloned Lin^neg^c-kit^pos^ CSCs respond *in vitro* to known cardiac morphogens like the Wnt/β-catenin and TGF-β/SMADs signaling pathways. Indeed, the canonical Wnt pathway, together with FGF and Hedgehog pathway, regulate cardiac progenitor cell proliferation in the mesoderm during embryonic life. On the contrary, Notch and non-canonical Wnt signaling regulate the differentiation processes during heart development (65–68). Interestingly, the cardiomyocyte differentiation program, in c-kit^pos^ CSCs, follows a step by step finely-regulated molecular cascade that is closely reminiscent of the known molecular program at the basis of the cardiac development from primary heart tube to the fetal/neonatal heart (54). *In vitro* administration of these specific cardiac morphogens allows to regulate the self-renewal potential and cardiomyogenic specification of CSCs to generate fully differentiated contracting CMs (52, 54, 69–72). CSCs express, Frizzled, the cell-surface receptor of Wnt/β-catenin canonical pathway, as well as its co-receptor, lrp-6, the low density lipoprotein receptor-related protein 6. Wnt-3a, Wnt-3a-conditioned medium, and bromoindirubin-3′-oxime (BIO) stimulate CSC expansion and clonogenicity, while canonical Wnt inhibition decreases CSC proliferation and clonogenicity *in vitro*. In contrast, Dickkopf-1 (Dkk-1) increases CSC myocyte specification, even though its effect is not sufficient to produce a fully differentiated contracting phenotype in culture. Additionally, clonogenic CSCs express TGF-β-R1, the cell surface receptor for TGF-β/SMAD signaling. In CM differentiation medium, BMP-2, BMP-4, TGF-β1, and Activin-A, factors that exert crucial roles in heart formation and CM specification during embryonic life, drive the expression of myogenic lineage markers in CSC culture increasing the number of cTnI^pos^ myocyte-committed cells (54, 73). Thus, CSCs respond to known cardiac morphogens. Inhibition of the Wnt canonical pathway and TGF-β family activation, each independently, promote cardiomyogenic commitment. Nevertheless, individual modulation of each of these cardiopoietic growth factors c(GFs) is insufficient to generate fully differentiated contracting CMs (74, 75). Remarkably, TGF-β family activation followed by the inhibition of the Wnt canonical pathway in a stepwise differentiation protocol induce full myogenic specification of CSC cultures with the appearance of spontaneously contracting cell clusters *in vitro* (52). Transcriptome comparison of RNA-seq data from CSCs, CSCs-derived CMs *in vitro*, neonatal CMs and adult CMs showed the highest similarity between CSCs-derived CMs and neonatal CMs. Therefore, the *in vitro* myogenic specification of clonogenic adult CSCs produces *bona fide* cardiomyocytes whose structural, molecular and functional maturity is nearly indistinguishable from neonatal mammalian cardiomyocytes (52, 54).

The regenerative capacity of adult endogenous CD45^neg^c-kit^pos^ CSCs has been evaluated using different rodent models of cardiac adaptations to stress and injury including diffuse myocardial damage inducing acute transient heart failure as well as physiological heart growth by exercise training (44, 76). After transplantation of *ex vivo* cloned and expanded c-kit^pos^ cardiac cells from old heart rodent donors, it was originally demonstrated that the infarcted myocardium showed the appearance of islands of regenerated cardiac muscle tissue, composed of new cardiomyocytes and microvasculature (22). Recently, this evidence has been independently reproduced showing that administering a cell progeny derived from a single CD45^neg^c-kit^pos^ clonogenic CSC genetically marked with GFP in syngeneic rats after experimental AMI, these cells provide robust histological and functional myocardial regeneration. At 28 days after AMI, CD45^neg^c-kit^pos^ clonogenic CSC GFP^pos^ revealed high engraftment rate in the border/infarct zone, yielding myocardial regeneration with formation of new cardiomyocytes, capillaries and arterioles. Furthermore, this regenerative effect was associated with reduced pre-existing CM apoptosis and hypertrophy, significantly decreased scar size and left ventricle dilation. All together these regenerative and cardioprotective effects improved cardiac function (52, 54). On the contrary, the administration of total c-kit^pos^ cardiac cells, which is mainly composed of CD31^pos^c-kit^pos^ endothelial committed cardiac cells, after AMI showed no regenerative nor cardioprotective effect on cardiac tissue histology and function with the detection of rare new cardiomyocytes. Most of the injected c-kit^pos^ total cardiac cells acquired endothelial lineage specification (52).

Overall, these data show that only ~1% of the total myocardial c-kit^pos^ population are real multipotent CSCs. The latter implies that c-kit is necessary but not sufficient to identify true adult CSCs. In order to assess the participation of CSCs in heart homeostasis/repair is therefore mandatory to identify this very small c-kit expressing regenerative population among the total c-kit^pos^ cardiac cells (54).

## c-kit Function in Endogenous CSC Biology and Cardiac Regeneration and Aging

**Figure 1:**
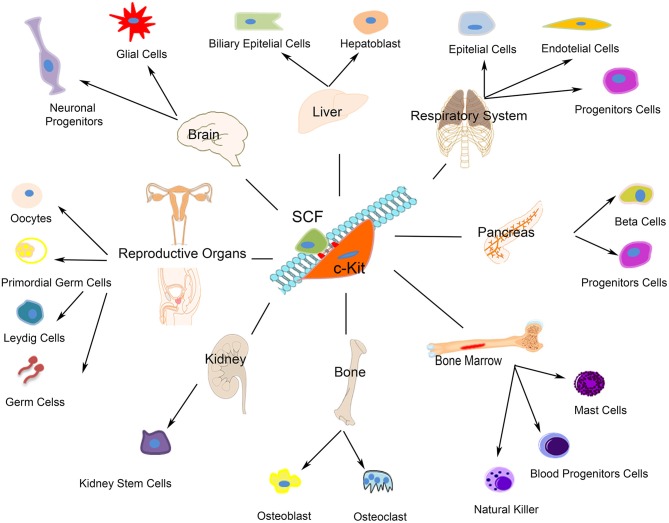
Schematic representation of c-kit expressing cells. The autophosphorylation of the c-kit receptor starts by the binding of its stem cell factor (SCF). As showed c-kit plays a significant role in the development of most organ systems such as neurons, interstitial cells of Cajal, hematopoietic progenitor cells, mast cells, Leydig cells, and spermatogonia.

While no single marker, including c-kit, exclusively identifies a cardiac stem cell, there is agreement that expression of c-kit, a type III tyrosine kinase receptor, marks a developmental stage between cardiac mesoderm formation and differentiation into the specific cardiovascular lineages (60, 77–79).

c-kit receptor's function depends from its phosphorylation that is started by the binding of the stem cell factor (SCF), which is expressed as a soluble or membrane bound splice variant. C-kit activation upon SCF trigger, which occurs either by the binding of free ligand or by heterotypic cell-cell interactions, modulates different cellular, and molecular programs including stem/progenitor maintenance, differentiation, proliferation, and migration in hematopoietic (80), germ (81), melanocyte (82), and other lineages [(83, 84); Figure 1]. Several forms of unregulated cell growth and tumor development depend from c-kit activating mutations (85). Undifferentiated as well as terminally differentiated cell types, such as neurons (86), interstitial cells of Cajal (87), hematopoietic progenitor cells (88), mast cells (89), and Leydig cells and spermatogonia (90) express c-kit on their membrane. Furthermore, c-kit plays a key role in the development of most organ systems, particularly in pigmentation, hematopoiesis, oncogenesis, and reproduction (Figure 1). Worth noting here that c-kit is a vital gene as indeed c-kit deletion in homozygosis is incompatible with life.

**Figure 2:**
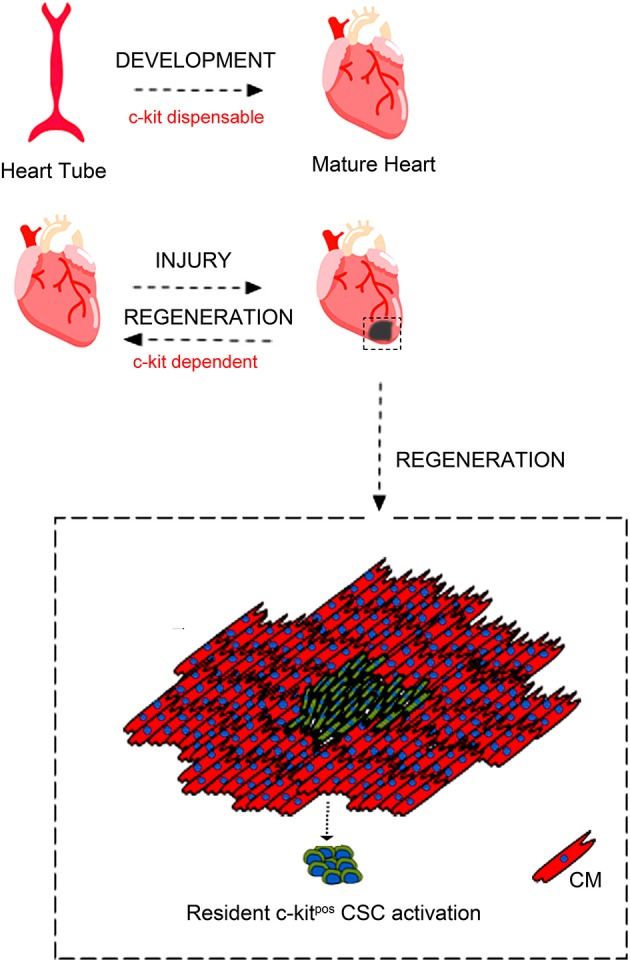
c-kit involvement in heart embryonic development and adult cardiac regeneration. During development c-kit expression appears dispensable for complete heart formation despite c-kit deletion is incompatible with life. Moreover, c-kit expression has been reported to be important also during heart cell specification and heart repair after injury through c-kit^pos^ CSCs activation. Adapted from Cianflone et al. (54) under the Creative Commons license CC BY-NC.

In regards with heart biology, c-kit expression has been shown in embryonic life during heart cell specification and it has been shown to play a role adult heart repair after injury [(86, 91); Figure 2].

To follow c-kit expression in cardiac cells, Tallini et al. (40) generated a BAC transgenic mouse (c-kit^BAC^-EGFP) in which the reporter gene, EGFP, is placed under the transcriptional control of the c-kit locus with the aim to have a reliable and easy-to-detect marker which maintains transcriptional fidelity. Using these c-kit^BAC^-EGFP transgenic mice, it was demonstrated that c-kit expression marks *bona fide* cardiac progenitor cells in the neonatal heart. Neonatal c-kit^pos^EGFP^pos^ cells are indeed able to differentiate *in vitro* into the cardiac cell lineages. Furthermore, when myocardial infarction is induced in these mice, a regenerative response is detected characterized by the recruitment of c-kit^pos^EGFP^pos^ cells, neomyogenesis and neoangiogenesis. Accordingly, these data were confirmed in human samples whereby it was shown that c-kit^pos^ cells are more abundant in the right atrial appendage of neonates while they decrease within the first month of life (92–94).

Mice lacking the receptor tyrosine kinase c-kit have hematopoietic defects causing perinatal death (95–97) and defective c-kit signaling leads to compromised cardiac function (98, 99); Figure 2]. Spontaneous murine genetic mutations in the c-kit locus determine the so called White (W) phenotype and heterozygous W mutant mice have an impaired c-kit signaling that is associated with worsened cardiac remodeling after MI (91). On the other hand, transgenic mice with c-kit over-expression mount an improved reparative response after MI leading to an increased cardiac function (54, 100, 101).

Recently, the function of c-kit signaling in CSC biology and heart repair, has been investigated (102) through a transgenic mouse model carrying an activated c-kit mutation. In particular, cardiac tissue and cardiac cells derived from these transgenic mice are characterized by a constitutive activation of the c-kit receptor (54, 102), which exerts a protective and regenerative role in myocardial tissue after injury. Stable c-kit activation improves cardiac remodeling and repair after myocardial injury while it fosters proliferation and differentiation of eCSCs mainly through MAPK and AKT signaling activation (54). Indeed, ERK1/2 and AKT phosphorylation, the molecular effectors of c-kit receptor downstream signaling, are significantly increased in heart tissue as well as in CSCs from the “*c-kit-activated”* transgenic mice (54, 102). These signaling pathways are instrumental in the modulation of the activation and endothelial/myogenic differentiation of CSCs (54, 102). These data overall show that c-kit receptor signaling modulates CSC fate *in vivo* and CSC endothelial as well as cardiomyocyte differentiation follows c-kit receptor molecular activation (54, 102).

If on one hand the positive modulation of c-kit receptor function positively modulates CSC regenerative potential, on the other, a c-kit null allele, as the one produced by Cre recombinase insertion in the c-kit locus to develop c-kit^Cre^-KI mice, offers a typical c-kit loss of function assay to assess whether eCSC function depends on an intact c-kit gene expression (54). Thus, we further tested the regenerative potential of Lin^neg^c-kit^pos^ CSCs obtained from c-kit^Cre^-KI mice compared with wild type Lin^neg^c-kit^pos^ CSCs (wtCSCs) before *in vitro* and then transplanting them *in vivo* in a murine myocardial infarction model (54, 103). We confirmed that Cre knock-in induced a typical White (W) mutation in c-kit^Cre^-KI mice that significantly reduced CSC proliferation and clonogenesis while nearly abolished the cardiomyogenic potential of these cells *in vitro* and *in vivo*. We then tested the effects of rescuing c-kit haploinsufficiency in c-kit^Cre^ CSCs by BAC-c-kit transgenesis. BAC-c-kit transfection normalized c-kit content in c-kit^Cre^ CSCs, which recovered a normal regenerative potential *in vitro* as well as *in vivo* after myocardial infarction (54, 103).

Therefore, the silencing of one c-kit allele, as resulting in a Cre knock-in mouse model, profoundly modifies c-kit biology and therefore this approach cannot be used to track c-kit expressing cells to study their physiology *in vivo*. An alternative approach to trace c-kit and evaluate its function in myocardial biology is the use of transgenesis to minimize all the deleterious effects of knock-in strategies. On this premise, Gude et al. generated a doxycycline-inducible transgenic mouse model to tag c-kit expressing cells with a long-lived, tetracycline responsive H2BEGFP (CKH2B) reporter (104, 105). In particular, they firstly confirmed that c-kit signaling promotes proliferation and survival of mouse and human cardiac progenitor cells while c-kit expression increases in response to cellular stress. The downstream effectors of c-kit phosphorylation, ERK and AKT, were coherently activated by SCF treatment and their activation was necessary for CPC activation *in vitro*. Furthermore, they compared the efficiency of identification of c-kit-labeled cardiac cells between the inducible-Cre knock-in line (c-Kit^MCM^) and the c-kit^H2BEGFP^ transgenic model, analyzing myocardial tissue sections from these two mutant mice. Interestingly, they found a higher density of c-kit^pos^ cardiac cells in c-kit^H2BEGFP^ transgenic vs. c-Kit^MCM^ hearts further demonstrating the negative impact of the Cre knock-in in the c-kit locus that turns in a significant reduction of cardiac c-kit-expressing cells (104). Furthermore, EGFP tagging of c-kit^pos^ cardiac cells was higher in c-kit^H2BEGFP^ transgenic vs. c-Kit^MCM^ hearts (104).

Overall, these data underline that genetic reporter and fate track mouse models are imperfect reproductions of endogenous gene expression. Indeed, both employing an exogenous promoter segment or exploiting the endogenous gene via knock-in strategy have limitations and caveats to be taken into account and precisely controlled for (106). Transgenic promoter segments may lack important regulatory elements possibly favoring ectopic expression, while knock-in reporters often create a null allele of the gene of interest with potential serious consequences on target cells. Specifically, applying knock-in strategy for c-kit-expressing cell lineage tracing creates a null allele of the c-kit gene. Indeed, Cre knock-in site disrupts known regulatory elements in exon 1, thereby perturbing endogenous c-kit biology with significant consequences for stem cell function (107). Additionally, reporter expression constrained to one allele of the endogenous promoter, coupled with decreased c-kit function, as it is the case of Cre knock-ins produce decreased reporter sensitivity and consequent under representation of the tagged c-kit cell population (19, 103, 108).

Several studies reproduced the findings that c-kit signaling promotes growth, survival and proliferation in human CPCs *in vitro* (109), while W locus mouse mutants (W/Wv) exhibit c-kit cell dysfunction (110, 111). W/Wv mice indeed display impaired cardiac recovery after infarction (98), diminished cardiac function with advanced age (99), and compromised c-kit cell differentiation into cardiomyocytes (99, 112). Bone marrow ckit^pos^ cells from W locus mutants or cells in which c-kit has been molecularly silenced *in vitro* exhibit blunted reparative responses to myocardial injury (91, 98). Furthermore, the deletion of c-kit gene, as it occurs in homozygous W-mutated mice (113), causes murine premature death, because c-kit gene deletion is incompatible with life. However, c-kit-defective adult hearts appear to develop normally during embryonic life (48), while adult c-kit^Cre^-KI mice have a significant defect in their regeneration potential after myocardial infarction *in vivo* (103). Therefore, it appears that c-kit plays divergent role in cardiac regeneration when compared to heart formation/development, which suggests that the molecular program underlying cardiac regeneration does not resemble cardiac generation. The latter is unpredicted when considering all the attempts currently ongoing to decode the pathways of developmental cardiac generation and neonatal heart regeneration to instruct effective protocols of adult cardiac regeneration (54).

Finally, the role of c-kit was evaluated in several models of cardiac pathology such as doxorubicin-induced cardiomyopathy (114–116), chronic heart failure (93, 117, 118), and aging cardiomyopathy (119, 120). In particular, Huang et al. developed a pediatric model of doxorubicin-induced cardiotoxicity in which juvenile mice were exposed to doxorubicin, using a cumulative dose that did not induce acute cardiotoxicity (114). These mice develop normally and have no obvious cardiac abnormalities as adults. However, these hearts have abnormal vasculature and a reduced number of c-kit^pos^ cardiac cells, which correlated with an increased sensitivity to physiological and pathological stimulus. When adult mice were subjected to myocardial infarction they developed a more pronounced cardiac decompensation, which correlated with a failure to increase capillary density in the injured area. Subsequently, it was demonstrated that the anthracycline-induced cardiomyopathy is caused by a depletion of functional c-kit^pos^ CSC pool and it can be rescued by restoring their function (115).

## Resolving the Controversy Over the Role and Myogenic Properties of the c-kit^pos^ CSCs

From the results summarized above, it was reasonable to expect that identification of the CSCs and characterization of their properties *in vitro* and *in vivo* would have put to rest any questions about the intrinsic regenerative capacity of the adult myocardium and about the origin of the CMs born in adulthood. Unfortunately, the notion that from a practical standpoint, the myocardium has neither intrinsic regenerative potential nor harbors tissue-specific stem cells with any meaningful myogenic capacities still persists (48, 121). This backwards view has persisted without a challenge to the reproducibility of published results which are the foundation of the new paradigm in heart biology (22, 32, 36, 37, 45, 52).

Putting aside the recent scandal over Anversa's group (see below), it remains the independently reproduced evidence arising from more than 15 years of scientific data (22). The burden of available scientific proof clearly shows that the adult mammalian heart harbors a pool of undifferentiated cells with cardiac regenerative potential, which are very small (5–7 μm in diameter), and are present in low abundance (1 CSCs per every (1–3) thousand CMs). Unsurprisingly, their identification, isolation and manipulation is naturally complex (53) as for the very nature of all adult tissue specific stem cells. *In vitro* and *in vivo* CSCs are *bona fide* myogenic progenitors, producing immature CMs of small size, which *in vitro* express cardiomyocyte-specific genes at levels similar to neonatal cardiomyocytes and *in vivo* undergo complete maturation over time, with terminal differentiation and permanent withdraw from the cell cycle (45, 52, 53).

On this premise, the detection in the healthy and pathological myocardium of a cohort of cells, which express myocyte-specific genes while still undergoing DNA replication should not be interpreted as evidence of adult cardiomyocyte un-expected division. Yet the sole identification of small mononucleated cells expressing CM-specific genes undergoing DNA replication and cytokinesis has been taken as sufficient proof that post-natal pre-exisiting cardiomyocyte division account for adult CM renewal, denying any contribution of CSC differentiation to new CM formation in adult cardiac tissue homeostasis and after injury (50, 122). Despite the latter, there is no confirmed evidence that mature and terminally differentiated CMs from any mammalian species can re-enter the cell cycle and undergo productive cytokinesis. All the so-called “pre-exisiting CM division” in adulthood occurs indeed in small-sized mononuclear CMs (122–124), while in rodents the vast majority of the adult CMs are mature-sized and bi-nucleated (125). Without any further evidence, the rare cases of cells expressing sarcomeric proteins while undergoing DNA replication and mitosis has recently been re-interpreted as evidence of a small pool of immature adult CMs which retain their proliferative competence (48, 122). This interpretation is based on the fact that in the neonatal life CMs, for a limited time window and before their terminal differentiation, can boost their replicative capacity and on the indisputable evidence that the CMs of certain fishes and amphibians are mitotically competent (126, 127). However, at present there is not a single piece of experimental evidence in support that these two phenomena have any relevance to CM renewal in the adult mammalian heart.

In contrast, no available data can dispute that the adult heart harbors resident CSCs and multiple laboratories have conclusively shown that these cells *in vitro* and *in vivo* generate *bona fide* cardiomyocytes together with vascular and connective tissue cells (30–35, 37, 45). Thus, the detection of dividing small, immature, and mono-nucleated CMs as found in the adult myocardium should be more appropriately interpreted as transient amplifying myocytes differentiated from a more resident stem/progenitor cell and surely not the proof of the division of pre-existing CMs (20, 128).

Recently several genetic murine approaches to track *in vivo* the so-called “c-kit^pos^ cardiac cells” (28, 29, 48–51, 121, 129) has generated a significant confusion calling for “a re-evaluation of the real myogenic potential of the cardiac c-kit^pos^ CSCs” (48).

Using either c-kit^Cre^-KI mice or c-kit^CreER^-KI mice, these authors reported that “cardiac c-kit^pos^ cells” mainly differentiate into endothelial cells and minimally, if not negligibly, contribute CMs either in neonatal or adult life, or after injury (48–50). According to these findings, it was claimed that the “cardiac c-kit^pos^ cells” are not CSCs at all but just endothelial committed cells (48, 49). Moreover, the regenerative potential of “cardiac c-kit^pos^ cells” is limited to neoangiogenesis and to cardiac interstitial cell formation (54).

To critically analyze the findings of these reports, it must be first remembered, as discussed above, that c-kit^pos^ cardiac cells are a heterogeneous cell population whereby in the adult heart >90% of c-kit^pos^ cells are mast cell/endothelial lineage-committed cells. Genetic fate mapping strategy base on the Cre-lox recombination system, nowadays considered “the gold standard” to address the exact regenerative potential of a given cell population, has been then customized to track the fate of c-kit-expressing cells *in vivo* (48–50). The latter was deemed sufficient by the proposed experimental design to include also c-kit-expressing CSCs. Unfortunately, all the insertions and deletions required to introduce the Cre recombinase into the c-kit locus have resulted in a null c-kit mutation, which does not produce the corresponding mRNA (103). Thus, these mice could be used only in heterozygosis while carrying a significant genetic defect with physiological consequences. Cre recombinase detects DNA sequences flanked by a specific 34-bp sequence called loxP removing the flanked sequence, and leaving single loxP site in place (130–133). This technology is used to delete a transcriptional stop sequence such that a reporter gene starts to be expressed after Cre recombination. The latter is the basis of the “indelible labeling” by Cre-lox-based lineage-tracing experiments. In these experiments, DNA excision at loxP sites is dependent on Cre expression whereby recombination occurs only in those cells that express or had expressed Cre recombinase. By placing the Cre cassette under the control of a specific gene promoter, recombination is directed to a particular cell expressing that particular gene. However, the mapping system is guided by Cre levels, whereby recombination efficiency is proportional to Cre levels. This is crucial because despite two cell types express the Cre-targeted gene but at different levels, not necessarily the two cell types will be equally recombined (54). If Cre levels efficiently recombine only one of the two cell types, the resultant fate map will underestimate the descendant population of the un-recombined cell type (54). Accordingly, the cells with lowest expression of Cre, because of the low expression of the Cre-engineered gene might fail to have their fate tracked (54, 133). In the case of c-kit fate tracking experiments, the Cre-dependent recombination efficiency is directly proportional to the level of Cre expression from the null c-kit allele (19, 133, 134). Considering that most stem cell types express low level of c-kit (54, 103, 135), and particularly c-kit^pos^ CSCs (54, 103), it was highly questionable whether the null c-kit^Cre^ allele could recombine a meaningful fraction of the c-kit^pos^ CSCs to track their fate (19).

Indeed, Vicinanza et al. (103) and Cianflone et al. (54) have recently shown that c-kit expression level in adult CSCs is low and the c-kit^Cre^ allele in c-kit^Cre^ KI mice produces insufficient amounts of Cre to effectively recombine the floxed Cre-reporter gene to tag the CSCs and fate their progeny (54, 103). Thus, c-kit^Cre^-KI models (48, 49) only minimally, if not negligibly, tag, and fate map resident CSCs. Furthermore, Cre-KI into c-kit locus in all cases has produced a null c-kit allele that fatally impairs *in vitro* and *in vivo* CSC properties (54, 103). This non-physiologic and inefficient recombination system, produced by the c-kit^Cre^-KI model, determines a very low number of c-kit^pos^ progenitor-generated cardiomyocytes detected in c-Kit^cre^ mice. This picture reflects the failure to recombine the CSCs to track their progeny and the severe defect in CSC myogenesis produced by the c-kit^cre^ allele (54, 103). For these reasons, unavoidably, all the c-kit^Cre^ knock-in mice show a scant CM progeny *in vivo* during homeostasis and after injury (48–50). Astonishingly, despite lacking proper controls (19) and despite the evidence for their severe limitations (103), the results arising from c-kit^Cre^ KI mice have been taken as evidence that c-kit^pos^ CSCs do not exist or have a marginal myogenic regenerative potential (136). Overall, while these papers have been proven wrong and unreliable, they have generated significant and unnecessary upheaval in the cardiac repair/regeneration field (19, 20).

To correctly track the fate of endogenous CSCs requires a c-kit-driven Cre KI mouse model that does not affect c-kit expression and in which Cre is produced in amounts sufficient to recombine the marker gene. Li et al. (137) attempted to overcome this problem using a dual reporter in which two loxP sites were interleaved so that either a Dre-rox or Cre-loxP recombination would remove the substrate of the other resulting in the permanent tagging of the cell. This system was used to label “all” CMs and non-CMs with two different markers. Surprisingly, they only ascertained that the “majority” of the cells were labeled but never tested whether this system was tagging the CSCs. Therefore, despite the controversy, they overlooked a basic rule of cell-fate tracking, which is that the marker used has to effectively tag the cell which fate is to be tracked. To assert that the system used tags most “non-myocyte” cells means very little, particularly when the population to be tracked (the cardiac stem/progenitor cells) represent <1% of the non-myocyte population which is labeled. Therefore, the main issue in the paper by Li et al. is that the authors never tested whether cardiac stem/progenitor cells were indeed labeled among the non-myocyte population. The authors did not carry out this essential step. Furthermore, another issue with Li et al. paper is that because they never tested if and how the adult CSCs were labeled, they cannot exclude the hypothesis that they were instead labeled by the myogenic/myocyte promoters they used in embryo life. The latter is a key step because it is known and proved that the Tnn2 promoter (used by Li et al.) during heart development in embryonic life not only labels cardiomyocytes but also endothelial cells covering aortic and pulmonary valves (138), a fact that indirectly shows that Tnn2 promoter activity in embryonic life labels multipotent cardiac progenitors. Additionally, adult cardiac stem/progenitor cells express Tnn2 mRNA *in vivo* (139, 140).

In short, despite contradictory publications, any objective review of the data shows that the eCSCs are genuine cardiac stem/progenitor cells and the main, if not the only, *bona fide* source of new cardiomyocyte formation in the healthy and pathological adult heart.

## Conclusions

The long-standing paradigm of the heart as a non-regenerative organ has been replaced by a wealth of data showing that new cardiomyocyte (CMs) are formed throughout life and after injury in the adult mammalian heart. It is also clear, however, that this regeneration on its own is not robust enough to repair severe segmental myocardial damage such as post-AMI, the main cause of HF. Overall, the data available show that when correctly identified and expanded the endogenous CSCs are robustly myogenic *in vitro* and *in vivo*. Unfortunately, despite this reproducible evidence, some recent work has questioned what was a growing consensus about the origin, quantity, and physiological significance of the CMs generated in adulthood in response to wear and tear and/or injury, which has severely muddled the field of adult cardiac regenerative biology. To move forward past this controversy is a crucial step for the adult cardiac regenerative biology field.

Very sadly and unfortunately, the recent scandal that brought to the request for and the retraction of a significant number of papers from the group of Piero Anversa, a group that historically was among the first to contribute to the discovery and characterization of adult cardiac progenitors, has created a “tsunami” for the field of adult cardiac stem cell biology (136). Clearly, scientific misconduct is a very serious issue which needs to be dealt seriously but honestly. It is understandable also that what has become public is sufficient reason to critically review Anversa's publications and the data which have been manipulated should be immediately retracted. However, it is mandatory that institutions, journals, and the scientific establishment in general will do that objectively and using the same parameters applied to others who have had publications retracted and/or discredited. Instead and very sadly, some investigators are using this turbulence to discredit all work related to myocardial repair/regeneration and cardiac stem cells, even though a significant part of the work which they now assail, has no relation to and was not authored by Anversa's group. Ironically, while arguing that, based on the revelations as to Anversa, all papers about myocardial repair/regeneration based on myocardial stem cells should be either ignored or retracted, these investigators fail to point out that the work providing negative data on this subject has been found incorrect and the relative methodology and conclusions have been shown to be invalid (103). In short, to call into a ban all the independent work produced by the cardiac stem cell field just on the basis of the proven and alleged Anversa's misdeeds is as unscientific as these misdeeds are.

It is clear that the c-kit^Cre^-KI strategies for CSC identification and cell-fate mapping have such severe limitations as to make them unsuitable for either the identification or fate-map the c-kit^pos^ CSCs. The very low number of endogenous c-kit^pos^CSC-generated cardiomyocytes detected in the c-kit^Cre^ mice does not reflect a minimal myogenic potential of the CSCs but it simply reflects the failure of the KI-Cre to recombine the CSCs to track their progeny together with the severe defect in CSC myogenesis produced by the c-kit^Cre^ null allele.

When the pitfalls of the c-kit^Cre^-KI are taken at a face value it follows that the results of the experimental approach though endogenous CSC ablation and their exogenous replacements clearly stand and indisputably show that the CSCs are necessary and sufficient for robust cardiomyogenesis and to support myocardial regeneration/repair in response to diverse types of damage. This phenotype requires and is dependent upon a diploid level of c-kit expression. Confirmation of these conclusions using novel and reliable genetic fate map strategies should clear the way for the potential development of CSC-based myocardial regenerative protocols.

## Author Contributions

DT, FM, VM, and BN-G have contributed to the conception or design of the work. MS, EC, TM, IA, VA, MT, and DP contributed to the acquisition, analysis, or interpretation of the data for the work. DT, FM, and BN-G drafted the work and revised it critically for important intellectual content. MS, EC, TM, IA, VA, DP, MT, and VM revised the work critically for important intellectual content. All the authors gave their final approval of the version to be published and agree to be accountable for all aspects of the work in ensuring that questions related to the accuracy or integrity of any part of the work are appropriately investigated and resolved.

### Conflict of Interest Statement

BN-G is the founder of company StemCell OpCo. The remaining authors declare that the research was conducted in the absence of any commercial or financial relationships that could be construed as a potential conflict of interest.
